# Identification and characterization of circRNAs related to meat quality during embryonic development of the longissimus dorsi muscle in two pig breeds

**DOI:** 10.3389/fgene.2022.1019687

**Published:** 2022-11-15

**Authors:** Jing Wang, Jun-Feng Chen, Qiang Ma, De-Lin Mo, Jia-Jie Sun, Qiao-Ling Ren, Jia-Qing Zhang, Qing-Xia Lu, Bao-Song Xing

**Affiliations:** ^1^ Henan Key Laboratory of Farm Animal Breeding and Nutritional Regulation, Institute of Animal Husbandry and Veterinary Science, Henan Academy of Agricultural Sciences, Zhengzhou, China; ^2^ State Key Laboratory of Biocontrol, School of Life Sciences, Sun Yat-sen University, Guangzhou, China; ^3^ Guangdong Provincial Key Laboratory of Animal Nutrition Control, Guangdong Laboratory for Lingnan Modern Agriculture, National Engineering Research Center for Breeding Swine Industry, College of Animal Science, South China Agricultural University, Guangzhou, China

**Keywords:** pig, circRNA, muscle development, lipid deposition, ceRNA

## Abstract

Meat quality, an important economic trait, is regulated by many factors, especially by genetic factors, including coding genes, miRNAs, and lncRNAs. Recent studies have elucidated that circRNAs also play a key role in muscle development and lipid deposition. However, the functions and regulatory mechanisms of circRNAs in meat quality remain mostly unknown. The circRNA expression profiles between Huainan pigs (Chinese indigenous pigs, fat-type, Huainan HN) and Large White pigs (Western commercial pigs, lean-type, LW) in the longissimus dorsi (LD) muscle at 38, 58, and 78 days post conception (dpc) were compared by sequencing. In total, 39,887 circRNAs were identified in 18 samples, and 60, 78, and 86 differentially expressed circRNAs (DECs) were found at the three stages mentioned above between these two breeds. The parent genes of DECs were enriched in myogenesis, proliferation, adipogenesis and muscle fiber-type transition. The circRNA-miRNA interaction networks included 38 DECs and 47 miRNAs, and these miRNAs were involved in muscle development and lipid metabolism. Two shared DECs (circ_0030593 and circ_0032760) of these three stages were selected, their head-to-tail junction sites were validated by Sanger sequencing, and RT‒qPCR results suggested that these two DECs might be involved in intramuscular fat deposition. These findings provide a basis for understanding the role of circRNAs in meat quality.

## Introduction

Pork is the predominant source of dietary protein worldwide. At present, the pork production can largely meet market’s demand, and higher requirements for pork quality has been put forward. Compared with the Western commercial pig breeds, Chinese indigenous pig breeds generally exhibit better meat quality, including bright meat color, thin muscle fibers and high intramuscular fat content. The differences in meat quality traits between Chinese indigenous breeds and Western commercial breeds provide good material for studying the genetic differences in meat quality traits. Screening new regulatory factors for meat quality is of great economic value to genetically improve meat quality traits.

A muscle fiber is the smallest functional unit of skeletal muscle, and its thickness, density, type and intramuscular fat content all influence meat quality. Muscle development involves a complex set of cellular and developmental processes that is regulated by many genes, transcription factors, and noncoding RNAs. Some studies have compared the expression of mRNAs, miRNAs and lncRNAs in different pig breeds at different developmental stages. For example, Cai et al. compared transcriptomic differences in longissimus dorsi (LD) muscle between Mashen (MS) and Large White (LW) pigs at 0, 90 and 180 days after birth and found that growth genes were associated with a faster growth rate in LW, while genes related to fatty acid synthesis were associated with higher intramuscular fat deposition in MS (Cai et al., 2020). Zhao et al. compared transcriptome differences between Lantang (LT) and Landrace (LR) pigs from 35 days post conception (dpc) to 180 days post-natal (dpn), confirming that 49–77 dpc is critical for muscle phenotype formation and that GSK3B may be involved in later myogenesis in LR, with some myogenic inhibitors also potentially contributing to the slower muscle differentiation rate in LT (Zhao et al., 2011). He et al. compared the expression of miRNAs in LD muscle of MS and LW at 35 dpc and identified 87 differentially expressed miRNAs, which were enriched in muscle contraction, WNT, mTOR, and MAPK (He et al., 2017). Herein, our team preliminarily compared lncRNA expression differences in LD muscle between Huainan (HN) and LW pigs at 38, 58, and 78 dpc. The results suggested that the most active period of muscle development between these two breeds was different, being more active at 58 dpc in the HN and 78 dpc in the LW. LncRNAs also participated in earlier myogenesis, shorter proliferation and higher intramuscular fat (IMF) deposition in HN. The PI3K/Akt and cAMP pathways are associated with IMF deposition (Wang et al., 2020a).

Circular RNA, having a covalent closed-loop structure, was first observed by electron microscopy in 1976, and by 2012, a large number of circRNAs had been discovered using high-throughput sequencing technology. Recent studies have shown that circRNAs are involved in the regulation of meat traits, such as circTAF8 (Li et al., 2021a), circCCDC91 (Zhao et al., 2022), circUBE2Q2 (Zhao et al., 2022), and circSVIL (Yue et al., 2022), which are involved in muscle development, and circPPARA (Li et al., 2022a), circINSR (Shen et al., 2020), and circ-ATXN2 (Song et al., 2021), which are associated with lipid deposition. Porcine circMYLK4 was also identified as a regulator of fast/slow myofibers (Cao et al., 2022). These studies have determined the functions of some circRNAs in different species, but studies on porcine circRNAs are limited, with most of them only performing differential expression profiling analysis and ceRNA network construction. For example, Hong et al. analyzed circRNA expression profiles in LD muscle of Duroc pigs at 33, 65 and 90 dpc and found circRNAs with higher expression levels at 33 dpc (Hong et al., 2019). Li et al. compared circRNA expression in LD muscle of Ningxiang pigs at 30, 90, 150 and 210 dpn and found that differentially expressed circRNAs (DECs) were enriched in muscle development and fatty acid biosynthesis signaling pathways (Li et al., 2021b). Jin et al. compared the whole transcriptional profiles of 47 different skeletal muscles in adult pigs and identified 48,232 circRNAs, elucidating the molecular regulatory differences in energy metabolism and contractile properties of different skeletal muscle sites (Jin et al., 2021). Wang et al. screened 66 DECs in the LD muscle between adult HN and Duroc×(Landrace×Yorkshire) (DLY) pigs that are involved in myogenesis, lipogenic differentiation and flavor through Wnt, the transition between fast and slow fibers, and alanine, aspartate and glutamate metabolism pathways (Wang et al., 2019a). Li et al. compared the expression of circRNAs in the LD muscle of MS pigs and LW pigs at 1, 90 and 180 days of age and screened 327 DECs enriched in TGF-β, MAPK, FoxO and other signaling pathways related to skeletal muscle growth and fat deposition (Li et al., 2021c).

In most species, the number of muscle fibers becomes fixed during the embryonic period, making this time critical for meat quality. Previous studies have indicated that primary muscle fibers are formed from 30 to 60 dpc, while secondary muscle fibers are formed from 54 to 90 dpc. Studies on the effect of porcine circRNAs on muscle development have made comparisons between different stages in the same pig breed or between different breeds in one developmental stage or different developmental stages after birth. However, circRNA expression differences in the LD muscle between fatty and lean pigs at embryonic stages have not been reported. HN pigs, an excellent indigenous Chinese pig breed, were included in “The fine local livestock and poultry breeds record of Henan province” in 1986 and are famous for their heat resistance, roughage resistance, large litter size, and particularly high intramuscular fat content. To date, the effect of circRNAs on muscle development in the embryo stage of Huainan pigs has not been reported. Therefore, RNA sequencing technology and bioinformatics methods were first applied to identify DECs in LD muscle between HN pigs (Chinese indigenous breed, fat type) and LW pigs (Western commercial breed, lean type) at different embryonic developmental stages, and functional validation and regulatory mechanism analysis were performed for shared DECs at different stages. The results of this study provide fundamental material for studying the mechanisms of circRNAs in porcine muscle development.

## Materials and methods

### Experimental animals and tissue collection

All pigs were fed by Henan Xing Rui Agricultural and Animal Husbandry Technology Co., Ltd (Henan Province, China). Five HN and five LW sows in their second or third parity were selected and artificially inseminated with sperm from the same breed (with the same genetic background). At 38, 58, and 78 dpc, one sow from each breed was slaughtered following national and institutional guidelines for the ethical use and treatment of animals in experiments. At 38 dpc, it was difficult to identify female and male fetuses by appearance, so all of the fetuses were immediately removed from the uteri and used for sample collection. The sexes of these fetuses were identified by the SRY gene, and then three male and three female fetuses were selected for sequencing. At 58 and 78 dpc, three male and three female fetuses were selected by appearance and used for sample collection. For all fetuses, the LD muscle tissue was collected from the same area and snap frozen in liquid nitrogen until further use. Existences and expressional levels of identified circRNAs were detected between eleven different tissues from adult HN pigs, including the heart, liver, spleen, lung, kidney, gut, stomach, LD muscle, subcutaneous adipose, intramuscular adipose, and abdomen adipose. The LD muscles were sampled from forty Duroc × (Landrace × Yorkshire) adult pigs with high IMF (>6%) and forty with low IMF (<3%), separately, and then the differences of the circRNAs’ expression between the high IMF pigs and low IMF pigs were detected.

### RNA isolation, quality control and library preparation

According to the manufacturer’s instructions, TRIzol reagent (Invitrogen Life Technologies, Carlsbad, United States) was used to isolate total RNA from LD muscle samples. For each stage of both breeds, one male RNA sample and one female RNA sample were mixed, and three mixed RNA samples were used for sequencing. A NanoDrop ND-1000 (Implen, Westlake Village, CA, United States), Agilent 2,100 Bioanalyzer (Agilent Technologies, United States) and denaturing agarose gel electrophoresis were used to test the purity, concentration and integrity, respectively, of the isolated RNA. Samples with high RNA integrity number (RIN) values (larger than eight) were used for library preparation. Ribosomal RNA was removed from the total RNA using the Ribozero™ rRNA Removal Kit (Epicenter, United States). Then, linear RNA was removed using an RNAse R kit (Epicenter, United States). The rRNA-free and linear RNA-free RNA was used to generate sequencing libraries with the NEBNext^®^ Ultra™ Directional RNA Library Prep Kit (NEB, Ipswich, MA, United States).

### CircRNA sequencing and circRNA identification

The Illumina HiSeqTM 2,500 platform (Novogene, Beijing, China) was used to sequence the generated libraries. TopHat2 software (v2.1.1) was used to map the clean data to the porcine reference genome (Sscrofa11.1). Find_circ algorithms (Rao et al., 2021) and CIRI2 (Gao et al., 2018) were used to identify circRNAs from the unmapped reads (read count ≥2). The expression levels of circRNAs are shown as transcripts per kilobase per million mapped reads (TPM). According to the mapping region to the annotated gene, circRNAs were classified as exonic, intronic, and intergenic circRNAs.

### Principal component analysis and hierarchical clustering

PCA of circRNA expression profiles was conducted using the R packages FactoMineR and Facto Extra. Hierarchical clustering of circRNAs was generated using the R package heatmap.

### Identification of DECs and analysis of their functional differences in circRNA expression

HN and LW animals at 38, 58, and 78 dpc were analyzed using the DEseq2 package (Love et al., 2014), and│log2foldchange│≥1 and *p*_adj_ ≤ 0.05 were used to identify DECs. DECs’ parental genes were subjected to Gene Ontology (GO) and Kyoto Encyclopedia of Genes and Genomes (KEGG) analyses using the DAVID tool (http://david.abcc.ncifcrf.gov/), and *p* < 0.05 was considered significant.

### DEC-miRNA network construction

MiRanda software (v3.3a) was used to predict potential binding sites of miRNA to DECs, as previously described (Wang et al., 2019b). The miRNAs were derived from the LD muscle between HN and LW at 38, 58, and 78 dpc, and these results have not been published before. Cytoscape (V3.2) software was used to visualize the potential DEC-miRNA regulatory network.

### Reverse transcription quantitative PCR

According to the manufacturer’s instructions, the Prime Script RT reagent Kit with gDNA Eraser (TaKaRa, Dalian, China) was used to convert total RNA to cDNA using random primers. A SYBR Green PCR kit (TaKaRa, Dalian, China) was used to perform qPCR. The outward-facing primers were used for circRNA identification. GAPDH was used as an internal control. All primers used for RT‒qPCR are shown in Table 1. Each qPCR experiment was performed in triplicate, and the fold-changes of circRNAs were calculated using the 2^−△△Ct^ method (Xu et al., 2017).

**TABLE 1:** Primers used for RT‒PCR.

Name	Sequence (5′-3′)	Size (bp)
circ_0030593	F: GTC​CGA​GGG​CAG​TGG​ACT​GG	123
	R: GGC​TTG​ATG​CAG​CAG​CAC​TT	
circ_0011630	F: AAT​GGC​ATT​TCC​AGG​AGG​TT	123
	R: TTG​TCA​GAC​TCC​ATG​GTA​CTT	
circ_0002895	F: GTG​ACA​TGG​AGT​CCA​TCA​TC	166
	R: ATG​ACG​AAT​TGA​ATT​CTG​CT	
circ_0032760	F: CTG​AAC​CAC​TGA​GCG​CTG​AG	121
	R: ACG​GCG​CAG​AGG​TCA​AGA​AG	
circ_0025881	F: GCA​AAA​CAC​ACT​CAG​ATG​AT	126
	R: CTT​GTC​CAT​TAA​TTC​GTT​CTT​C	
circ_0028985	F: CCA​TCG​ATA​TCC​AGG​TTG​TTG​AG	129
	R: TCA​GTG​ATG​CCG​TAC​TGG​AA	
PPAR gamma	F: AGG​ACT​ACC​AAA​GTG​CCA​TCA​AA	142
	R: GAG​GCT​TTA​TCC​CCA​CAG​ACA​C	
FABP4	F: ATG​AAA​GAA​GTG​GGA​GTG​G	156
	R: ATC​AAA​TTC​CTG​GCC​CAA​TT	
AdipoQ	F: CGA​TTG​TCA​GTG​GAT​CTG​ACG	151
	R: CAA​CAG​TAG​CAT​CCT​GAG​CCC​T	
GLUT4	F: TAA​GAC​AAG​ATG​CCG​TCG​GG	136
	R: GAG​AAG​ACG​GCG​AGG​ACA​AG	
ADD1	F: CGA​TTC​GCC​CCT​GAG​AAC​AC	129
	R: CTG​GGA​CCA​TTC​AGC​CTC​TC	
GAPDH	F: ACC​AGG​TTG​TGT​CCT​GTG​AC	94
	R: AGC​TTG​ACG​AAG​TGG​TCG​TT	

### Culture and differentiation of porcine intramuscular adipocytes

Following previously described methods (Chen et al., 2017), intramuscular adipocytes were isolated from porcine LD muscle and cultured in normal medium, defined here as DMEM/F12 supplemented with 10% FBS and antibiotics (100 IU/ml penicillin and 100 μg/ml streptomycin) at 37°C and 5% CO_2_ (Day 0, D0).

Differentiation was induced as follows: differentiation medium I (“normal medium” supplemented with 5 μg/ml insulin, 1 mM DEX, and 0.5 mM IBMX) was changed 48 h after 100% confluence (Day 2, D2). Two days later, differentiation medium II (“normal medium” supplemented with 5 μg/ml insulin) was changed (Day 4, D4). Two days later, the normal medium was changed (Day 6, D6).

### CircRNA overexpression

Circ_0030593 was amplified to construct the pCD2.1-circ_0030593 overexpression vector (Geneseed, Guangzhou, China). When the intramuscular adipocytes reached 70%–80% confluence, the pCD2.1-circ_0030593 and pCD2.1 empty vectors were transfected using DharmaFect 2 (Dharmacon, Lafayette, CO, United States) at a final concentration of 7 μL/mL, and the medium was changed 12 h later. Overexpression efficiency was assessed 24 h after transfection. Four days (D4) and six days (D6) after differentiation, the cells were harvested for RT‒qPCR of several adipogenesis marker genes and Oil Red O staining.

### Statistical analysis

The R statistical package (version 3.6.1) was used to analyze the data. The results are presented as the mean ± standard error, and a *p*-value <0.05 was considered statistically significant.

## Results

### Basic features of CircRNAs

**FIGURE 1:**
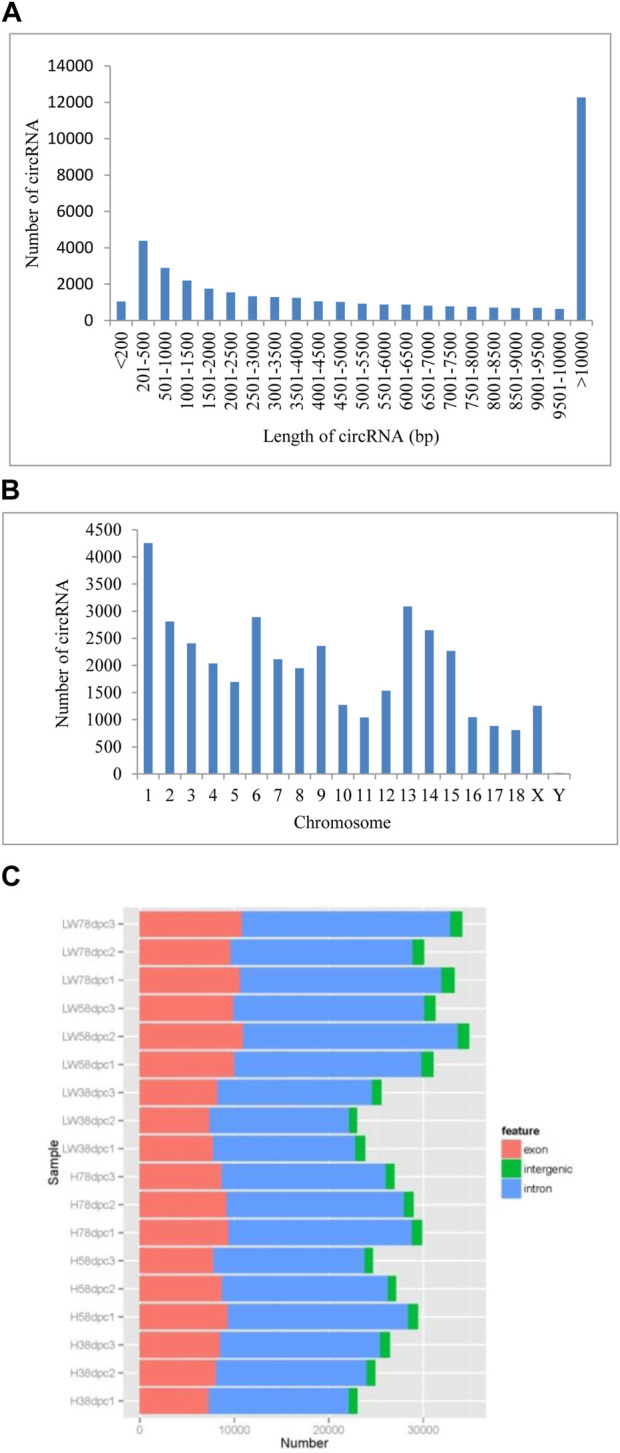
The genomic features of novel circRNAs in LD muscle of HN and LW at different embryonic periods **(A)** Length distribution of novel circRNAs. **(B)** Chromosome distribution of novel circRNAs. **(C)** Classification of the novel circRNAs.

A total of 39,887 circRNAs were identified from 18 samples. The length of the circRNAs ranged from 150 to 98,866 nucleotides (nt), 30.87% of which were larger than 10,000 nt, and for circRNAs less than 10,000 nt, those with lengths of 200–500 comprised the highest percentage (Figure 1A). These circRNAs were distributed across all chromosomes, with chromosome one having the most circRNAs (4,255) and chromosome Y having the fewest circRNAs (only 18) (Figure 1B). Based on chromosome location, these circRNAs can be classified into exonic, intergenic and intronic circRNAs. In each sample at each stage in both breeds, the most circRNAs were located in intronic regions, and the fewest circRNAs were located in intergenic regions (Figure 1C).

### Differential expression of circRNA between HN and LW pigs

**FIGURE 2:**
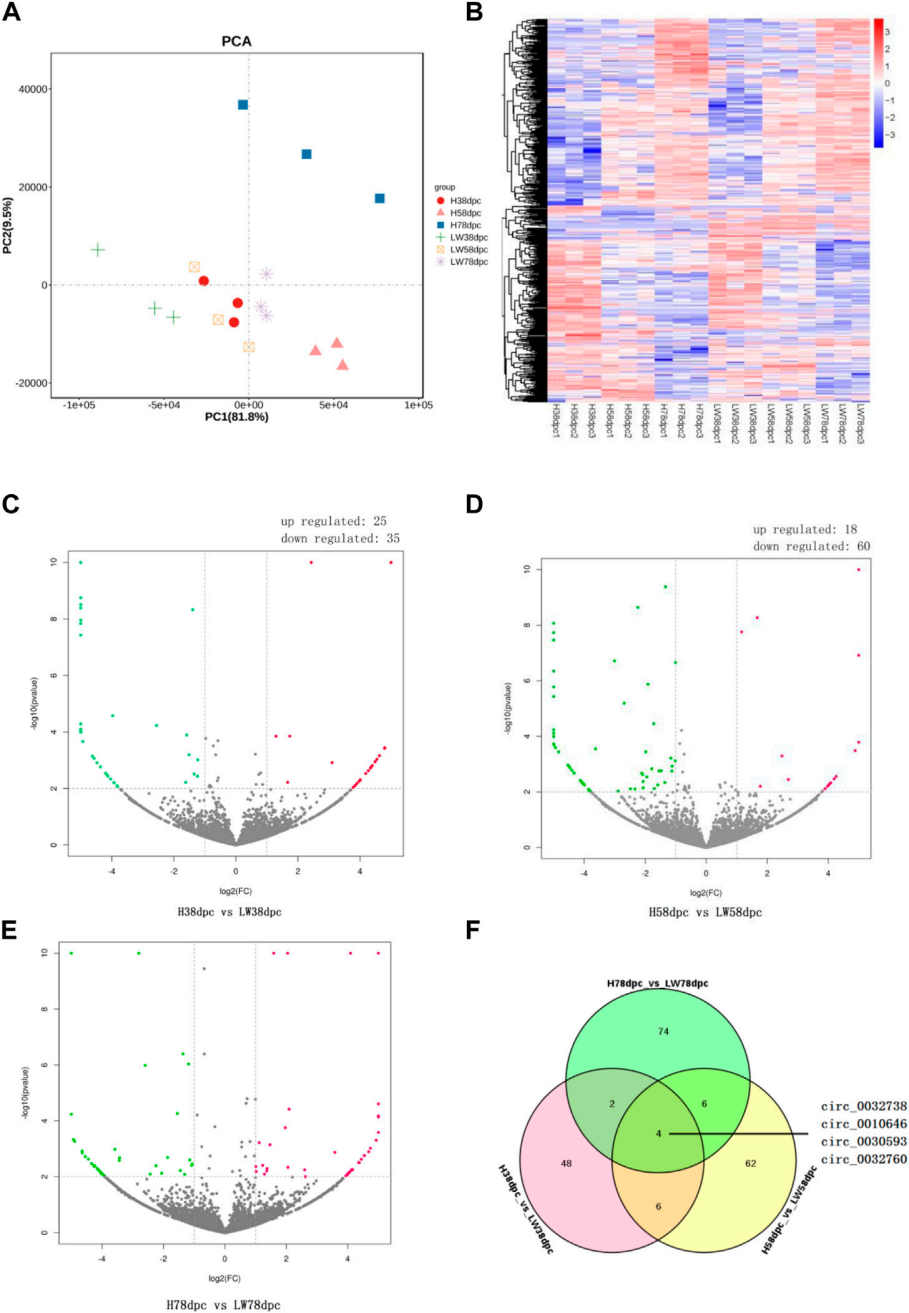
Principal component analysis (PCA) plot **(A)** and heatmap **(B)** of all detected circRNAs from 18 samples. Volcano plots of differential circRNAs between HN and LW pigs at 38 dpc **(C)**, 58 dpc **(D)**, and 78 dpc **(E)**. **(F)** Venn diagrams of DECs between HN and LW at different stages.

As shown by PCA (Figure 2A) and the heatmap (Figure 2B), the 18 samples clustered together at the same stage in the same breeds, and it is evident that the genetic relationship of three samples of HN78dpc are far away from other samples, followed by LW38dpc and H58dpc, while nine samples of LW58dpc, H38dpc, and LW78dpc were closer together. Comparing these two breeds, the number of DECs for the three stages were 60, 78 and 86, respectively, and in each stage, there were more downregulated DECs than upregulated DECs (Figures 2C–E). There were four shared DECs in all three stages: circ_0032738, circ_0010646, circ_0030593, and circ_0032760 (Figure 2F). Compared these four shared DECs with our previous results between HN and DLY, and it was found that the expressional difference of circ_0010646 and circ_0030593 between HN and DLY were also significantly, and both of these circRNAs showed lower expressional level in HN (Wang et al., 2019a).

### Functional enrichment analyses of DECs

**FIGURE 3:**
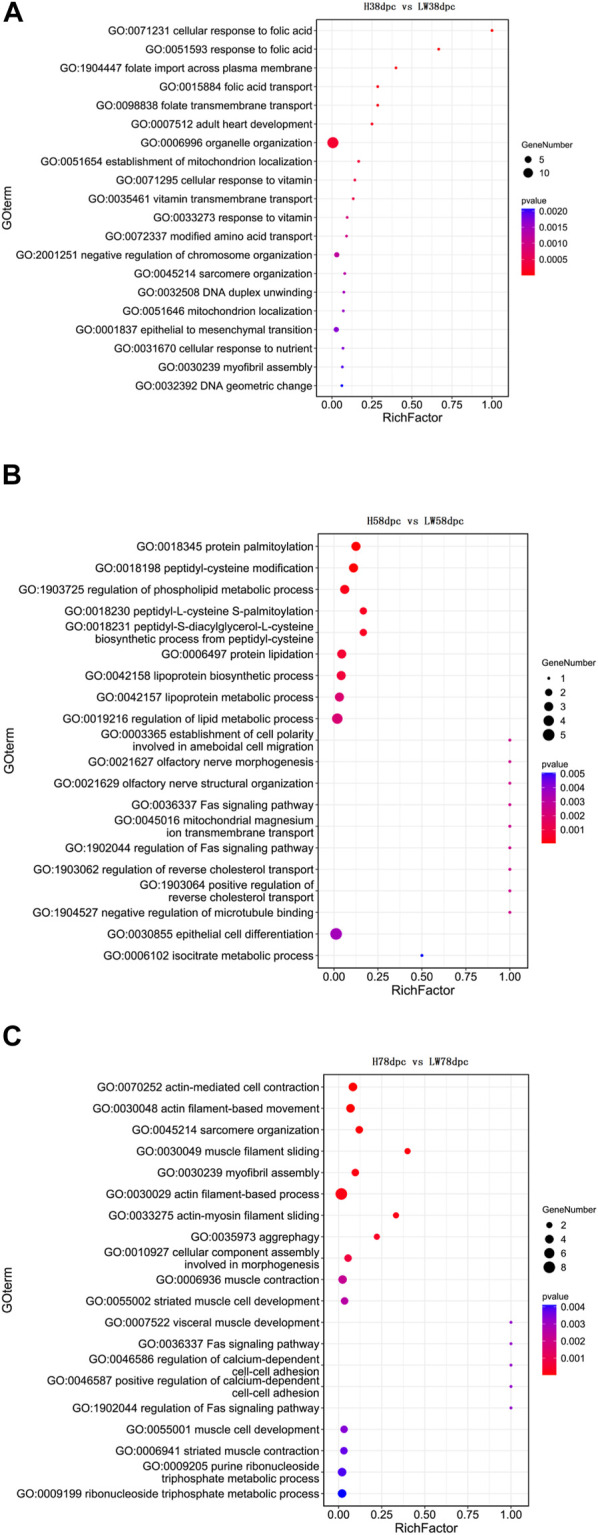
GO enrichment analyses based on parent genes of differential circRNAs at 38 dpc **(A)**, 58 dpc **(B)**, and 78 dpc **(C)**.

The GO results indicated that at 38 dpc, the parent genes of DECs were mainly enriched in the transportation of folic acid, vitamins, and the location of mitochondria and muscle development-related pathways, such as adult heart development and myofibril assembly (Figure 3A). In 58 dpc, synthesis, metabolism, and regulation of lipoprotein were enriched, along with cholesterol transport, cell differentiation and Fas pathways (Figure 3B). At 78 dpc, the genes were mainly enriched in muscle development-related pathways, such as muscle filament sliding, myofibril assembly, actin filament-based process, muscle contraction, striated muscle cell development, muscle cell development, and striatal muscle contraction, in addition to the Fas pathway (Figure 3C).

**FIGURE 4:**
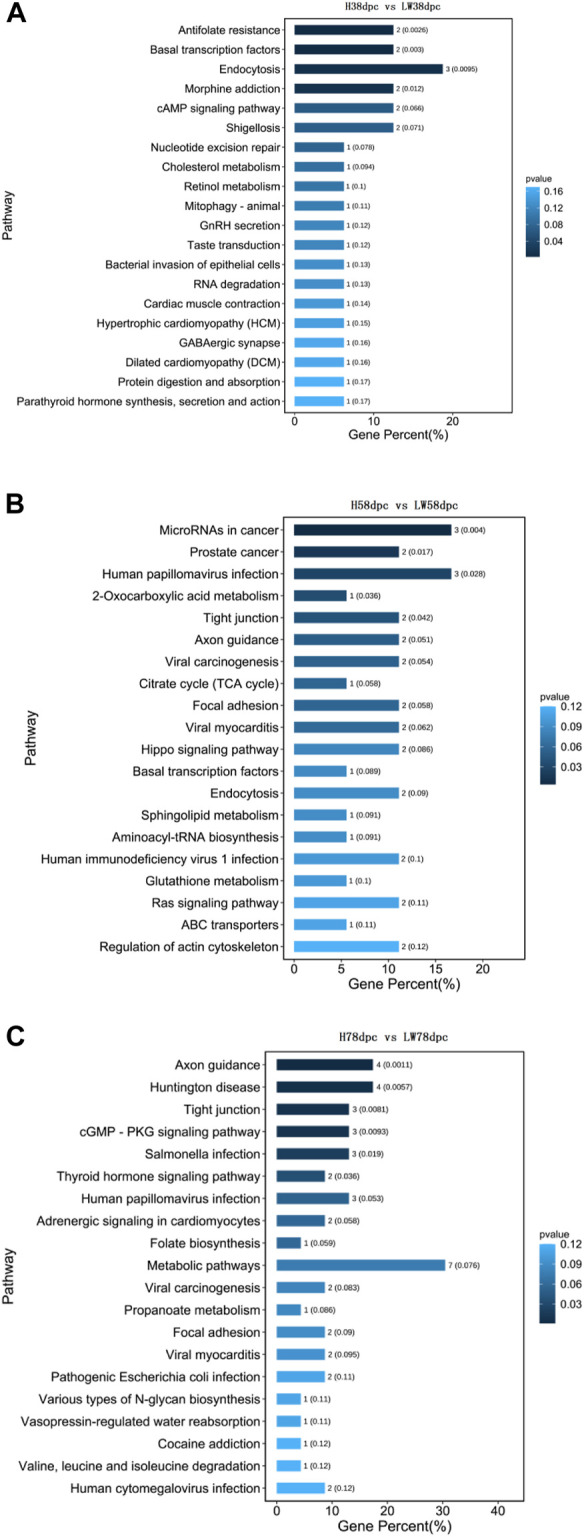
KEGG pathway analysis based on parent genes of differential circRNAs at 38 dpc **(A)**, 58 dpc **(B)**, and 78 dpc **(C)**.

As shown in Figure 4, the KEGG results indicated that muscle development-related pathways were enriched in different stages, involving hypertrophic cardiomyopathy (HCM), cardiomyopathy (DCM), viral myocarditis dilation, adrenergic signaling in cardiomyocytes, and cardiac muscle contraction. Meanwhile, adipose differentiation- and lipid deposition-related pathways were also enriched, such as PI3K-Akt, cAMP, Wnt, type I diabetes mellitus, cholesterol metabolism, cGMP-PKG, sphingolipid metabolism, and the TCA cycle. In addition, muscle fiber type transition-related pathways were enriched, such as AMPK, Wnt, and thyroid hormone.

### DEC-miRNA network construction

**FIGURE 5:**
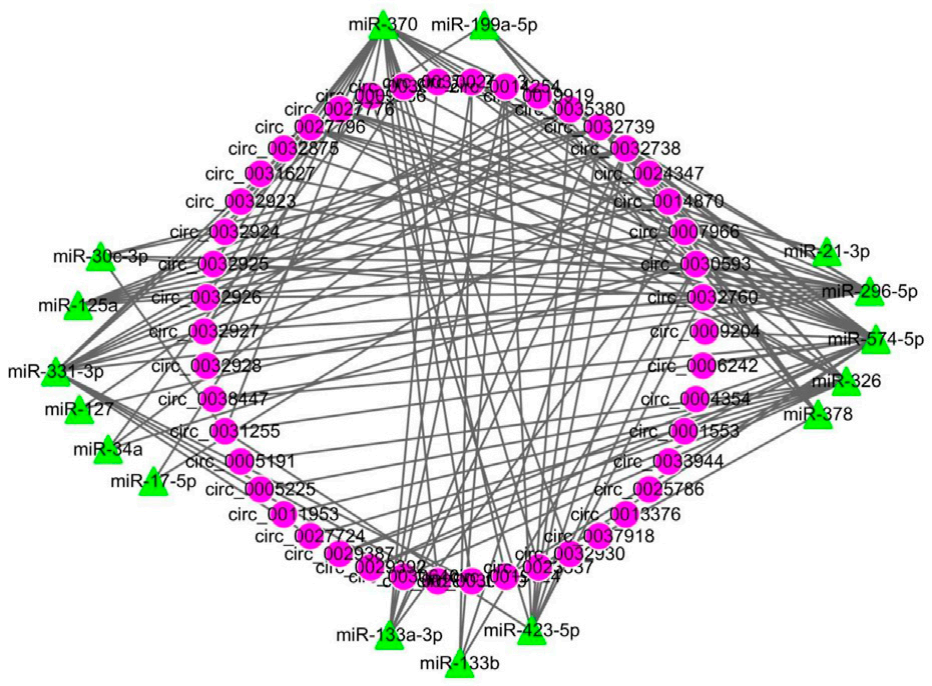
The circRNA-miRNA network of all differentially regulated circRNAs between HN and LW at 38 dpc, 58 dpc, and 78 dpc. Circular nodes represent circRNAs; triangular nodes represent miRNAs.

All DECs in the three stages in HN and LW pigs were subjected to interaction analysis with miRNA (Figure 5). A total of 38 DECs corresponding to 47 miRNAs were selected, and their interactions were made into a network with a total of 271 edges, of which miR-4331-3p corresponded to the most circRNAs (36 circRNAs), while circ_0035380 had the most target miRNAs (27 miRNAs).

### Validation of cric_0030593 and circ_0032760

**FIGURE 6:**
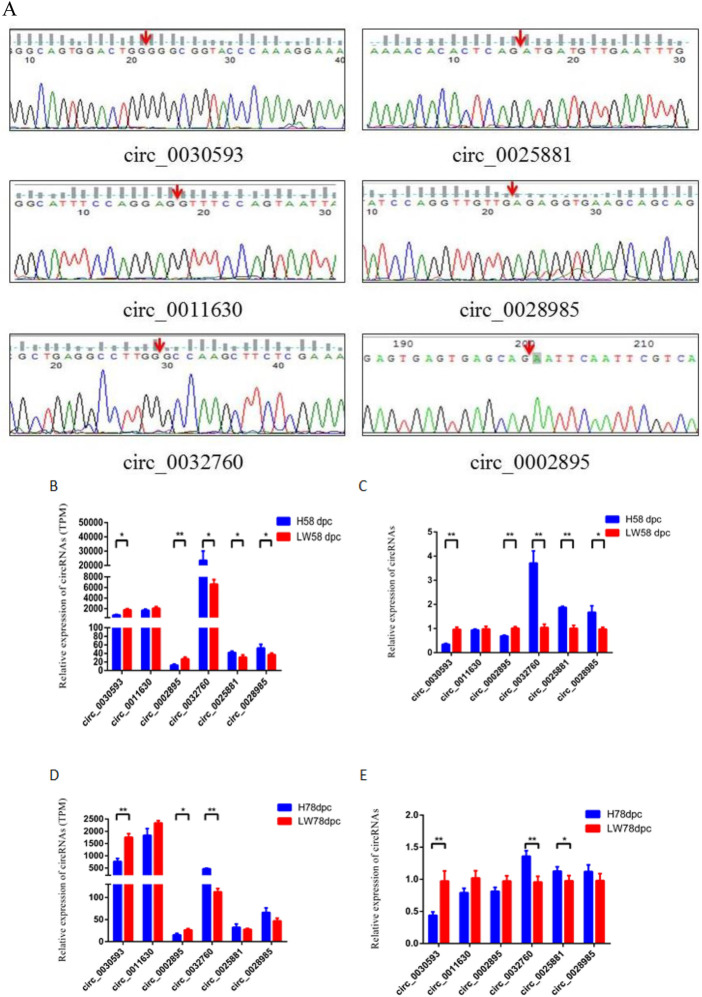
Validation of differently expressed circRNAs **(A)** The back-splicing junction sequence of circRNAs by sanger sequencing using the convergent primers. RNA sequecing **(B,D)** results and RT-qPCR results **(C,E)** of six differently expressed circRNAs between HN and LW at 58 dpc and 78 dpc.

**FIGURE 7:**
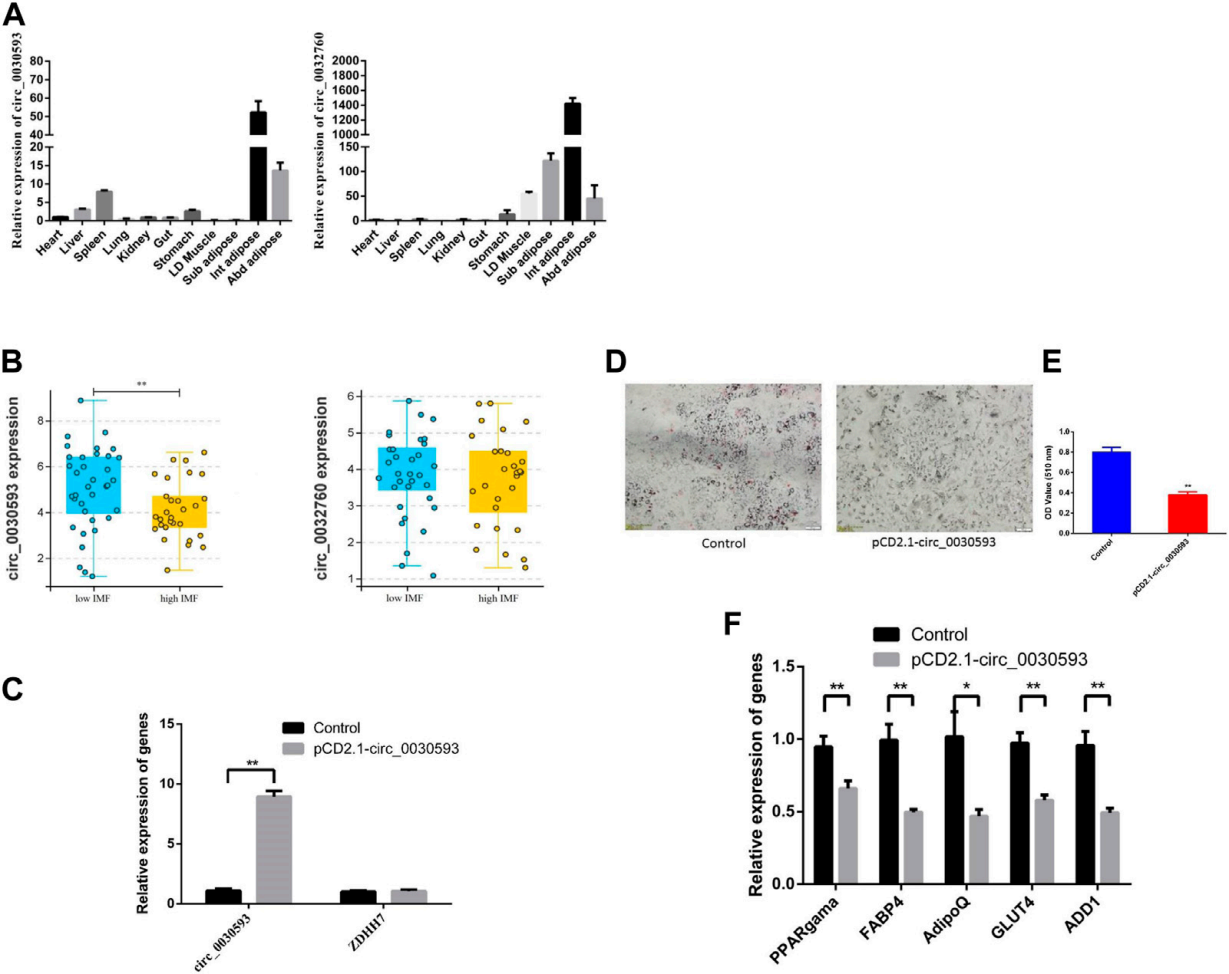
Function validation of differently expressed circRNAs **(A)** The expression level of circ_0030593 and circ_0032760 in different tissues by RT-qPCR. **(B)** circ_0030593 and circ_0032760 expression in longissimus dorsi muscle from high IMF and low IMF pigs **(C)**The effect of pCD2.1-circ_0030593 on expression of circ_0030593 and its parent gene ZDHH7. Overexpression of circ_0030593 inhibited adipogenesis by Oil Red O staining **(D)**, OD value **(E)** and RT-qPCR **(F)**.

The junction sites of circ_0030593, circ_0025881, circ_0011630, circ_0028985, circ_0032760 and circ_0002895 were confirmed by PCR amplification and sequencing (Figure 6A). The RT‒PCR results revealed that these circRNA expression patterns were consistent with the RNA-seq results (Figures 6B–E). Two shared DECs were subjected to tissue expression profiling, and circ_0030593 was most highly expressed in intramuscular adipose and subcutaneous adipose tissues (Figure 7A). Circ_0032760 was most highly expressed in adipose tissues, with the highest concentration found in intermuscular adipose tissue, followed by muscle tissue (Figure 7A). It was found that both circRNAs showed highest expressional levels in intramuscular adipose tissue, indicating that these two DECs might play an important role in intramuscular fat deposition, so we firstly verified their regulation role in adipogenesis of porcine intramuscular adipocytes. To further evaluate the role of circ_0032760 and circ_0002895 in IMF deposition, a potential correlation with IMF content of LD muscle was examined in another cohort comprising 40 DLY pigs with high IMF and 40 with low IMF. The results revealed that circ_0030593 exhibited higher expression levels in the low IMF group than in the high IMF group (*p* < 0.01), but the expressional difference of circ_0032760 between these two groups was not significant (*p* > 0.05) (Figure 7B). Further overexpression of circ_0030593 in porcine primary intramuscular adipocytes, the expression of circ_0030593 was significantly upregulated, but the expression of its parental gene ZDHH7 was not affected (Figure 7C). And oil red O staining suggested that elevated expression of circ_0030593 had an inhibitory effect on lipid deposition (Figures 7D,E). The RT‒qPCR results suggested that upregulation of circ_0030593 inhibited lipogenic differentiation, and the expression of PPAR gamma, FABP4, AdipoQ, GLUT4 and ADD1 was downregulated (Figure 7F).

## Discussion

Pork is the most commonly consumed meat in the world, and pork production has largely met the demand. Currently, consumers are increasingly interested in meat quality, so screening for key regulatory factors related to meat quality has significant economic value. Many studies have identified meat quality-related circRNAs, such as those regulating myoblast proliferation and differentiation (circMYBPC1 (Chen et al., 2021), circ-FoxO3 (Li et al., 2019), and circEch1 (Huang et al., 2021)) and those regulating fat deposition (circINSR (Shen et al., 2020), circ-PLXNA1 (Wang et al., 2020b), and circPTK2 (Ding et al., 2021)). Most studies on porcine circRNAs have only performed differential expression analyses, such as screening DECs in LD muscle between obese and lean pigs at one developmental stage (Wang et al., 2019b; Li et al., 2021c; Li et al., 2022a) or screening DECs in different muscle fiber types (Li et al., 2020; Jin et al., 2021). In contrast, research about specific DECs’ molecular regulatory mechanisms is limited; however, it was reported that circTUT7 regulates HMG20B expression through miR-30a-3p and thus affects embryonic development of skeletal muscle (Hong et al., 2019). Furthermore, circPPARA promotes intramuscular adipogenesis *via* miR-429 and miR-200b (Li et al., 2022a). Therefore, this study compared circRNA expression in LD muscle between HN and LW pigs at different embryonic stages (38, 58, and 78 dpc) and performed functional enrichment and ceRNA regulatory network analysis of DECs. In addition, a preliminary analysis of the molecular regulatory mechanisms of shared DECs at different stages was performed. Considering the animal welfare and economic cost, in current study 3 litters were taken from the same sow, genetically unrelated litters could be sampled to further validate the conclusions in this study. But the results of the current study still provide some material for understanding the role of circRNAs in meat quality.

Most circRNAs regulate expression of their parental gene, which in turn affects related traits. Therefore, in this study, DECs’ parental genes were subjected to GO and KEGG analysis. Previous studies have shown that there are significant differences in embryonic muscle development between Chinese indigenous pig breeds and Western commercial breeds. Zhao et al. compared embryonic developmental differences in LD muscle between LT (obese) and LR (lean) breeds and found that the primary fibers emerged at 35 dpc and 49 dpc, respectively. At 49 dpc, secondary muscle fibers could be detected in both breeds, but at 91 dpc, LT had fewer muscle fiber numbers and smaller muscle fiber diameters (Zhao et al., 2011). Similarly, the number and density of myoblasts in TC were greater than those in YK pigs at 30 dpc (Zhao et al., 2015). These studies indicate that during the embryonic period, compared to Western commercial pig breeds, Chinese indigenous pig breeds exhibit earlier myogenesis, shorter proliferation, and consequently lower meat production. In the present study, many muscle development-related signaling pathways were enriched, with HCM and DCM being consistently upregulated in HN. The transcription and metabolism of folic acid were enriched at 38 dpc, and it was reported that folic acid is necessary for the proliferation and differentiation of myoblasts (Hwang et al., 2018). In contrast, the Wnt pathway, which promotes myogenic differentiation (Zhou et al., 2015), was significantly upregulated at both 38 and 58 dpc. These results are consistent with earlier myogenic differentiation and proliferation in Chinese indigenous pig breeds. Rap1 (Li et al., 2018), adhesion junctions (Ghosh et al., 2022), tight junctions (Kusch et al., 2009), ras (Tyurin-Kuzmin et al., 2020) and other signaling pathways associated with myoblast proliferation and muscle development were downregulated at 58 dpc and/or 78 dpc. These results suggest that at 58 dpc and 78 dpc, muscle development in the HN was less active than that in the LW, consistent with previous findings that Chinese indigenous pig breeds exhibit slower muscle development than Western commercial breeds during the later embryonic period (Zhao et al., 2011). This result is also consistent with our previous studies on lncRNAs (Wang et al., 2020a).

It has been reported that during the later embryonic development period, intramuscular fat deposition in Chinese indigenous pigs is different from that in Western commercial breeds (Zhao et al., 2015). In the present study, multiple pathways associated with adipose differentiation and lipid deposition were enriched at different stages, such as the cAMP pathway, which inhibits adipogenesis and promotes lipolysis (Liu et al., 2017), was upregulated at 38 dpc and downregulated at 78 dpc. The cGMP/PKG pathway, which promotes adipogenesis (Chen et al., 2007), displayed higher expression at 78 dpc. The estrogen pathway, which inhibits IMF deposition (Renaville et al., 2012), was downregulated at both 38 and 78 dpc. The Fas signaling pathway, which is involved in intramuscular fat deposition (Cui et al., 2012), was enriched at 58 and 78 dpc. Other pathways associated with lipid metabolism, such as type I diabetes mellitus, cholesterol metabolism, and sphingolipid metabolism, were upregulated in HN at different developmental periods. These results coincide with the higher IMF content in Chinese indigenous pig breeds.

Muscle fiber type also affects meat quality, such as meat color and tenderness (Lee et al., 2010). The proportion of muscle fiber types was found to be different between Chinese and Western commercial pig breeds (Kunej et al., 2005; Huang et al., 2016). In this research, AMPK exhibited lower expression in HN at 78 dpc, and Wnt displayed higher expression levels in HN at 38 and 58 dpc. Both of these pathways can promote a fast-to-slow fiber type shift (Tee et al., 2009; Liu et al., 2016). The thyroid hormone pathway, which affects muscle fiber type, was also enriched (Salvatore et al., 2014).

CircRNAs can also function as miRNA sponges. For example, circ-FoxO3 inhibits C2C12 myoblast cell differentiation by sponging miR-138-5p (Li et al., 2019). CircINSR inhibits preadipocyte adipogenesis by regulating Foxo 1 and EPT 1 *via* miR-15/16 (Shen et al., 2020). CircHIPK3 promotes the proliferation and differentiation of myoblasts by sponging miR-7 (Gao et al., 2021). CircFUT10 promotes adipocyte proliferation *via* the let-7-PPARGC1B (peroxisome proliferator-activated receptor *γ* coactivator 1-*β*) pathway (Jiang et al., 2020). In the current study, the DEC-miRNA network included 62 nodes (44 circRNAs and 16 miRNAs) and 141 edges, of which miR-370 had the most target circRNAs (23 circRNAs) and circ_0032738 had the most miRNA binding sites (12 miRNAs). Therefore, these DECs might participate in the regulation of meat quality by sponging these miRNAs.

To validate the RNA sequencing results, 6 circRNAs were confirmed by amplification with convergent primers. Expression of these circRNAs at the 58 dpc and 78 dpc stage was analyzed by RT‒qPCR, and the results were consistent with the sequencing results. To determine how these circRNAs regulate muscle development or fat deposition, two shared DECs (circ_0030593 and circ_0032760) were selected for functional verification. Both circRNAs showed the highest expression levels in intramuscular adipose tissue, indicating that these two DECs might play an important role in intramuscular fat deposition, so we first verified their regulatory role in the adipogenesis of porcine intramuscular adipocytes. The expression difference of circ_0030593 between the high and low IMF group was significant (*p* < 0.05), suggesting that circ_0030593 may have an inhibitory effect on intramuscular fat deposition. Further overexpression experiments in porcine primary intramuscular adipocytes confirmed that elevating circ_0030593 inhibited lipid deposition. However, the mechanism of circ_0030593 is still not entirely clear. Circ_0030593 contains binding sites for many miRNAs associated with adipogenesis and lipid deposition, such as the miRNAs promoting adipogenesis and lipid deposition (miR-574-5p (Li et al., 2022b), miR-326 (Feng et al., 2020), miR-296-5p (Cazanave et al., 2011), and miR-378 (Podkalicka et al., 2022)) and miRNAs inhibiting lipid deposition (miR-125a (Xu et al., 2018), miR-127 (Gao et al., 2019), miR-34a (Wang et al., 2020c), miR-199a-5p (Alexander et al., 2013; Shi et al., 2014), and miR-370 (Chu et al., 2021; Zhang et al., 2021)). Therefore, circ_0030593 may be involved in regulating lipid deposition and thereby affecting intramuscular fat deposition by binding these miRNAs, but the specific miRNA or miRNAs need to be verified using dual luciferase, RIP and ChIRP assays in future research.

In conclusion, circRNA expression differences in LD muscle between HN and LW pigs at different embryonic stages were compared, and a DEC-miRNA network was constructed. The results indicated that circRNAs participate in the regulation of embryonic muscle development differences between HN and LW by regulating myogenesis, proliferation, adipogenesis and muscle fiber transformation. We also identified a novel circRNAs circ_0030593, that showed the highest expression levels in intramuscular adipose tissue, and in primary intramuscular adipocytes, it inhibited lipid deposition. These results provide basic material for understanding the effect of circRNA on meat quality.

## Data Availability

The datasets presented in this study can be found in online repositories. The names of the repository/repositories and accession number(s) can be found below: https://www.ncbi.nlm.nih.gov/, SRP243554.
